# RanBP9 Overexpression Accelerates Loss of Pre and Postsynaptic Proteins in the APΔE9 Transgenic Mouse Brain

**DOI:** 10.1371/journal.pone.0085484

**Published:** 2014-01-14

**Authors:** Hongjie Wang, Ruizhi Wang, Shaohua Xu, Madepalli K. Lakshmana

**Affiliations:** 1 Section of Neurobiology, Torrey Pines Institute for Molecular Studies, Port Saint Lucie, Florida, United States of America; 2 Department of Biological Sciences, Florida Institute of Technology, Melbourne, Florida, United States of America; University of Sydney, Australia

## Abstract

There is now compelling evidence that the neurodegenerative process in Alzheimer’s disease (AD) begins in synapses. Loss of synaptic proteins and functional synapses in the amyloid precursor protein (APP) transgenic mouse models of AD is well established. However, what is the earliest age at which such loss of synapses occurs, and whether known markers of AD progression accelerate functional deficits is completely unknown. We previously showed that RanBP9 overexpression leads to robustly increased amyloid β peptide (Aβ) generation leading to enhanced amyloid plaque burden in a mouse model of AD. In this study we compared synaptic protein levels among four genotypes of mice, i.e., RanBP9 single transgenic (Ran), APΔE9 double transgenic (Dbl), APΔE9/RanBP9 triple transgenic (Tpl) and wild-type (WT) controls. We found significant reductions in the levels of synaptic proteins in both cortex and hippocampus of 5- and 6-months-old but not 3- or 4-months-old mice. Specifically, at 5-months of age, rab3A was reduced in the triple transgenic mice only in the cortex by 25% (p<0.05) and gap43 levels were reduced only in the hippocampus by 44% (p<0.01) compared to wild-type (WT) controls. Interestingly, RanBP9 overexpression in the Tpl mice reduced gap43 levels by a further 31% (p<0.05) compared to APΔE9 mice. RanBP9 also further decreased the levels of drebrin in the hippocampus by 32% (p<0.01) and chromogranin in the cortex by 24% (p<0.05) compared to APΔE9 mice. At 6-months of age, RanBP9 expression in the cortex led to further reduction of rab3A by 30% (p<0.05) and drebrin by 38% (p<0.01) compared to APΔE9 mice. RanBP9 also increased Aβ oligomers in the cortex at 6 months. Similarly, in the hippocampus, RanBP9 expression further reduced rab3A levels by 36% (p<0.01) and drebrin levels by 33% (p<0.01). Taken together these data suggest that RanBP9 overexpression accelerates loss of synaptic proteins in the mouse brain.

## Introduction

Alzheimer’s disease (AD) is a devastating neurodegenerative disease of elderly that affects more than 35 million people worldwide [1]. AD is characterized by gradual intellectual deterioration and behavioral disturbances throughout the course of the disease. Accumulating data suggest that the progression of AD is more tightly associated with synapse degeneration rather than amyloid plaques or neurofibrillary tangles. For example, substantial evidence indicates that in AD, there is a decrease in the number of synapses, which occurs later than Aβ accumulation and correlates with disease progression [2]–[4]. Consequently, AD has been suggested to be a form of synaptic plasticity failure [5]. Ultrastructural analysis of autopsied brain tissue from patients with AD within few years after clinical onset revealed progressive synapse loss in the hippocampus, the frontal and inferior parietal cortex and entorhinal cortex [6], [7]. Even in cases of mild AD, as much as 55% loss of synapses has been reported within the hippocampus [7]. The role of synaptic proteins, especially their progressive loss in causing dementia has been the subject of increasing interest ever since the correlation between loss of synapses and AD was first established [8]. Biochemical analysis further showed that both the presynaptic protein synaptophysin [9] and the synaptic membrane and postsynaptic proteins such as synaptobrevin and synaptopodin [10], [11] are severely altered in the brains of patients with AD. However, as of today the molecular pathways responsible for either the synapse loss or differential vulnerability is not clear and therefore understanding the cellular and molecular mechanisms responsible for synaptic damage is critical for designing future therapeutic strategies for AD.

Loss of synapses has also been confirmed in several mouse models of AD [12]–[15]. In a most recent study, a transgenic mouse model with knockin expression of human mutant APP and/or human presenilin showed significant loss of synaptophysin-immunoreactive presynaptic boutons in the CA1-2 region of hippocampus at 10-months of age [16]. In APP/PS1 double transgenic mice (APΔE9), the density of large spines in plaque-free regions of the dentate gyrus is significantly reduced at 12–14 months of age coincident with impairment of cognition [17]. Thus, animal models provide a good opportunity to test the temporal sequence of synaptic protein loss. Despite abundant evidence for loss of synaptic proteins and cognitive impairment, it is not clear precisely when the earliest alterations occur in mouse models of AD. It is also unknown whether known Alzheimer’s risk factors accelerate loss of synapses and synaptic proteins. Thus, determining the earliest onset of memory deficits has been one of the main challenges in cognitive studies of AD. This is an important issue because identifying molecules that cause memory deficits depends upon accurately determining when cognitive deficits first appear. Transgenic mouse models provide excellent opportunities to test the effect of risk factors.

RanBP9 is a scaffolding protein that integrates a variety of signals from cell surface receptors to the intracellular targets [18], [19]. RanBP9 is known to exist and function in multiprotein complexes [20], [21]. We previously demonstrated that RanBP9 forms tripartite protein complex by binding with APP, BACE1 and low-density lipoprotein receptor-related protein (LRP), thereby increases Aβ generation in both transformed cells and primary neurons by enhancing β-secretase-mediated processing of APP at the cost of α-secretase processing [22]. Subsequently we confirmed increased amyloidogenic processing of APP by RanBP9 *in vivo*, by documenting increased amyloid plaque burden in a mouse model of AD [23]. Because RanBP9 protein levels are increased in the brains of patients with AD [24] as well as APP transgenic mouse models [25], [26], increased Aβ levels and associated pathology in AD is at least partly due to RanBP9. In line with this hypothesis, RanBP9 was recently found to be within the clusters of RNA transcript pairs associated with markers of AD progression [27], suggesting that RanBP9 might actually contribute to the pathogenesis of AD. In fact, we recently confirmed an inverse relationship between RanBP9 levels and spinophilin, a marker of spines in the synaptosomes of Alzheimer’s brains. RanBP9 overexpression in the APP/PS1 (APΔE9) mouse model was also accompanied by significantly impaired learning and memory skills [28]. These multiple evidences strongly suggest that RanBP9 plays pivotal role in the synaptic damage in AD. However, it is not clear whether RanBP9 overexpression exacerbates synaptic damage and if so whether it anticipates loss of synaptic proteins to earlier ages. Therefore identifying synaptic marker changes that follow cognitive deficits in early AD is critical as the accompanying synaptic changes can be effectively targeted by current treatments. Also detecting synaptic protein changes before the cognitive deficits appear have enormous implications for preventive and prophylactic treatments.

Here to get an overall picture of synaptic damage, we examined the levels of two presynaptic proteins, gap-43 and rab-3a and two postsynaptic proteins, drebrin A and chromogranin B at the earliest ages. Rab3a is a small vesicle protein while chromogranin B is a component of large dense core vesicles, all used as an estimate of synaptic density. GAP-43 is a component of presynaptic membranes while drebrin is a neuron specific major F-actin-binding protein abundantly found in dendritic spines. These four proteins together would represent the synaptic machinery including not only the small and large synaptic vesicles but also the pre and postsynaptic membranes. The levels of these proteins directly reflect number of synapses and since they play crucial role in synaptic plasticity, quantifying their protein levels is very important.

## Materials and Methods

### Chemicals and Antibodies

Protease inhibitor cocktail (cat # P8340), sodium orthovanadate (cat # S6508) and dithiothreitol (cat # D9779) were purchased from Sigma Aldrich (St. Louis, MO, USA). Microcystin-LR (cat # 475815) was obtained from Calbiochem (La Jolla, CA, USA). Polyclonal chromogranin antibody (cat # ab12242) was purchased from Abcam (Cambridge, MA, USA). Monoclonal anti-drebrin antibody (D029-3) was purchased from MBL international corporation (Woburn, MA, USA). Polyclonal Rab3A antibody (15029-1-AP) was purchased from ProteinTech Group Inc. (Chicago, IL, USA). Rabbit polyclonal antibody against Gap-43 was obtained from Millipore (Temecula, CA, USA). Anti-flag tag antibody (M2; F3165) was purchased from Sigma Aldrich (St. Louis, MO, USA). Polyclonal Aβ oligomer antibody, clone A11 (cat# AHB0052) was obtained from Life Technologies (Grand Island, NY, USA). Mouse monoclonal antibody against beta-actin (cat # A00702) was purchased from Genscript USA Inc. (Piscataway, NJ, USA). Secondary antibodies such as peroxidase-conjugated AffiniPure goat anti-mouse (Code # 115-035-146) and ant-rabbit (code # 111-035-144) IgGs were purchased from Jackson ImmunoResearch Laboratories (West Grove, PA, USA).

### Mice

All animal experiments were carried out based on ARRIVE guidelines and in strict accordance with the National Institute of Health’s ‘Guide for the Care and Use of Animals’ and approved by the Torrey Pines Institute’s Animal Care and Use Committee (IACUC). Generation of RanBP9 transgenic mice have been described previously [23]. The RanBP9 specific primers used in the polymerase chain reaction (PCR) is as follows. The forward primer is 5′ – gcc acg cat cca ata cca g -3′, and the reverse primer is 5– tgc ctg gat ttt ggt tct c –3′. Positive mice were then backcrossed with native C57Bl/6 mice and the colonies were expanded. RanaBP9 transgenic line 629 was used to breed with B6.Cg-Tg, APPswe, PSEN1ΔE9 (APΔE9) mice for generating triple transgenic mice (APΔE9/RanBP9). We obtained APΔE9 from Jackson Labs (Bar Harbor, Maine, USA). These double transgenic mice express a chimeric mouse/human APP (Mo/HuAPP695swe) and a mutant human presenilin 1 (PS1-ΔE9) both directed to CNS neurons. These APΔE9 transgenic mice were generated by co-injection of APP695swe and PS1-ΔE9 encoding vectors controlled by their own mouse prion protein promoter element. These mice were backcrossed to maintain C57Bl/6 background, expanded and genotyped to confirm the transgene using the following primers. The forward primer is 5′ – gac tga cca ctc gac cag gtt ctg –3′ and the reverse primer is 5 - ctt gta agt tgg att ctc ata tcc g –3′. Only male mice were used for all genotypes. These mice were used to measure synaptic protein levels by standard immunoblots. The number of mice, 6 for the WT and RanBP9 transgenic mice and 8 for the APΔE9 and APΔE9/RanBP9 mice were based on both statistical power analysis and our own previous experience on the same parameters.

### Tissue Extraction and Immunoblotting

The mouse brain tissues from four different genotypes, viz., wild-type (WT), RanBP9 single transgenic (Ran), APΔE9 double transgenic (Dbl) and APΔE9/RanBP9 triple transgenic (Tpl), all in C57BL6 background were dissected on ice immediately after euthanasia to obtain cortex and hippocampus tissue. Brain lysates were prepared from 3-, 4-, 5- and 6-months-old male mice from all four genotypes. In brief, we anesthetized the mice with isoflurane, decapitated them immediately and rapidly removed the brain tissues in to 1% NP40 buffer (50 mM Tris-HCl, pH 8.0, 150 mM NaCl, 0.02% sodium azide, 400 nM microcystine-LR, 0.5 mM sodium vanadate and 1% sodium Nonidet P-40) containing complete protease inhibitor cocktail for use with mammalian cell and tissue extracts (Sigma, St. Louis, USA). To extract Aβ oligomers RIPA buffer with SDS was used. Tissue was homogenized using Power Gen 125 (Fisher Scientific, Pittsburgh, USA) and centrifuged at 100,000 g for 1 h in a Beckman ultracentrifuge. Protein concentrations from each sample were measured in duplicates by BCA method (Pierce Biotechnology Inc., Rockford, USA). Before loading on to gels, the lysates were mixed with loading buffer containing dithiothreitol. SDS-PAGE electrophoresis was done exactly as published [22]–[24]. Briefly, Equal amounts of proteins were loaded into each well and subjected to electrophoresis. To separate Aβ oligomers, NuPAGE gels 4–12% were used. The proteins were then transferred onto PVDF membranes, blocked with 5% milk and incubated overnight with primary antibodies followed by one hour incubation with HRP-conjugated secondary antibodies such as monoclonal mouse anti-Goat IgG light chain or monoclonal mouse anti-Rabbit IgG light chain. The protein signals were detected using Super Signal West Pico Chemiluminescent substrate (Pierce, USA). Quantification of Western blot signals was done using imageJ software.

### Immunohistochemistry

Brain sections (16 um) from 6-month-old WT and APΔE9/RanBP9 triple (Tpl) transgenic mice were washed with PBS 1X 3 times each for 5 min. Antigen retrieval was carried out by immersing slides in 10 mM citric acid (pH 6.0) for 10 min at 90–95°C. Sections were washed with PBS 1X for 5 min 3 times, and incubated in blocking solution (10% normal goat serum, 1% BSA, 0.1% Triton X-100 in PBS 1X) for 1 h at room temperature. The sections were incubated overnight with specific antibodies against rab3a, gap43, drebrin and chromogranin in blocking solution (1∶200) at 4°C. After washing in PBS 1X for 5 min 3 times, the sections were incubated with Alexa Fluor® 568 goat anti-mouse IgG (Invitrogen) in blocking solution (1∶500) at room temperature for 2 h in the dark. Finally, slides were washed with PBS for 5 min 3 times, covered with mounting medium for fluorescence with DAPI (Vector Laboratories) and sealed with nail clear. Sections were visualized in a confocal microscope (Nikon C1Si laser scanning multispectral confocal microscope). Images of frontal cortex and CA1 region of hippocampus were obtained at 20x and processed using Image-Pro Plus (Media Cybernetics) software package. Positive immunoreactive stainings for all four synaptic proteins were observed in the neurons whose nuclei were co-localized with DAPI.

### Statistical Analysis

Immunoblot signals for rab3A, gap43, drebrin A and chromogranin B were quantified using publicly available Java-based ImageJ software. Levels of Aβ oligomers in APΔE9 versus APΔE9/RanBP9 mice were analyzed by Student’s paired t-test considering two-tailed P value for significance. The protein levels in WT, Ran, APΔE9 and APΔE9/RanBP9 mice were analyzed by one-way analysis of variance (ANOVA) followed by Tukey-Kramer multiple comparison post-hoc test for comparisons among WT, Ran, APΔE9 and APΔE9/RanBP9 mice at different ages for interaction effects using Instat3 software (GraphPad Software, San Diego, CA, USA). The data presented are mean±SEM. The n = 6 for WT and Ran genotypes and n = 8 for the APΔE9 and APΔE9/RanBP9 genotypes. The data were considered significant only if the p<0.05, * indicates p<0.05, **, p<0.01 and ***, p<0.001.

## Results

### RanBP9 Accelerates Loss of Presynaptic Proteins, rab3A and gap43 in the Cortex and the Hippocampus

**Figure 1:**
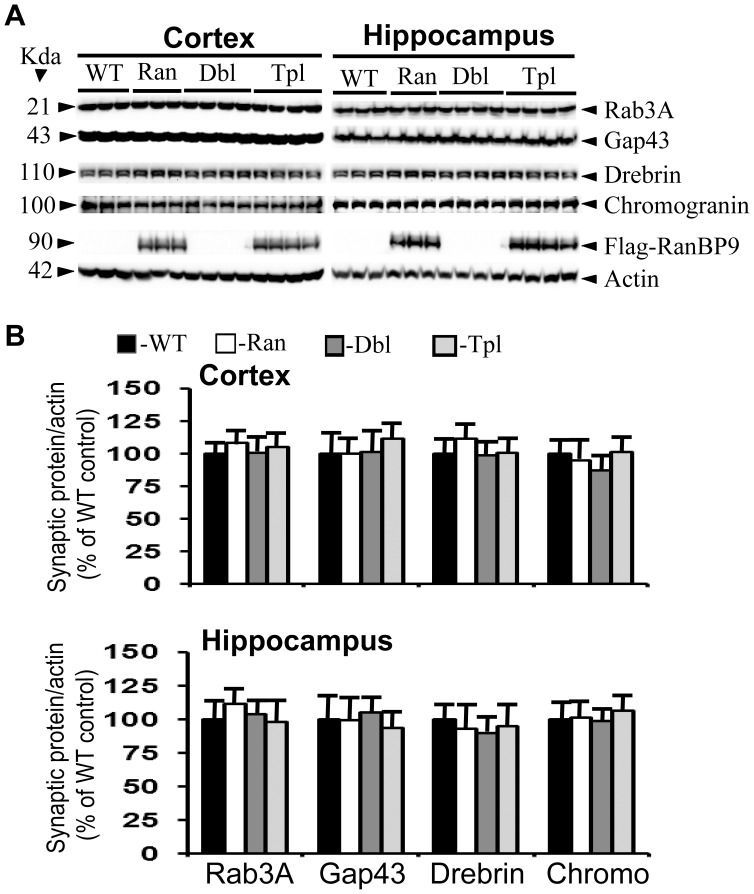
RanBP9 overexpression does not alter synaptic protein levels in the cortex and hippocampus at 3- months of age. **A:** Shows an immunoblotting analysis of rab3A, gap43, drebrin, chromogranin and the house keeping gene actin in brain samples from cortex and hippocampus. Brain homogenates from RanBP9 transgenic (Ran), APΔE9 double transgenic (Dbl), APΔE9/RanBP9 triple transgenic (Tpl) and age-matched wild-type (WT) control mice at 3-months of age were subjected to SDS-PAGE electrophoresis and probed with their respective antibodies. Flag specific monoclonal antibody detected flag-tagged exogenous RanBP9 in the RanBP9 single transgenic and APΔE9/RanBP99 triple transgenic mice only. Actin was used as loading control. The numbers on the left side indicate the molecular weights of each protein. **B:** Image J quantitation and normalization to actin levels showed no changes in the levels of any of the synaptic proteins at 3 months. The data are mean±SEM, n = 6 for WT and RanBP9 single transgenic, and n = 8 for APΔE9 and APΔE9/RanBP9 genotypes.

**Figure 2:**
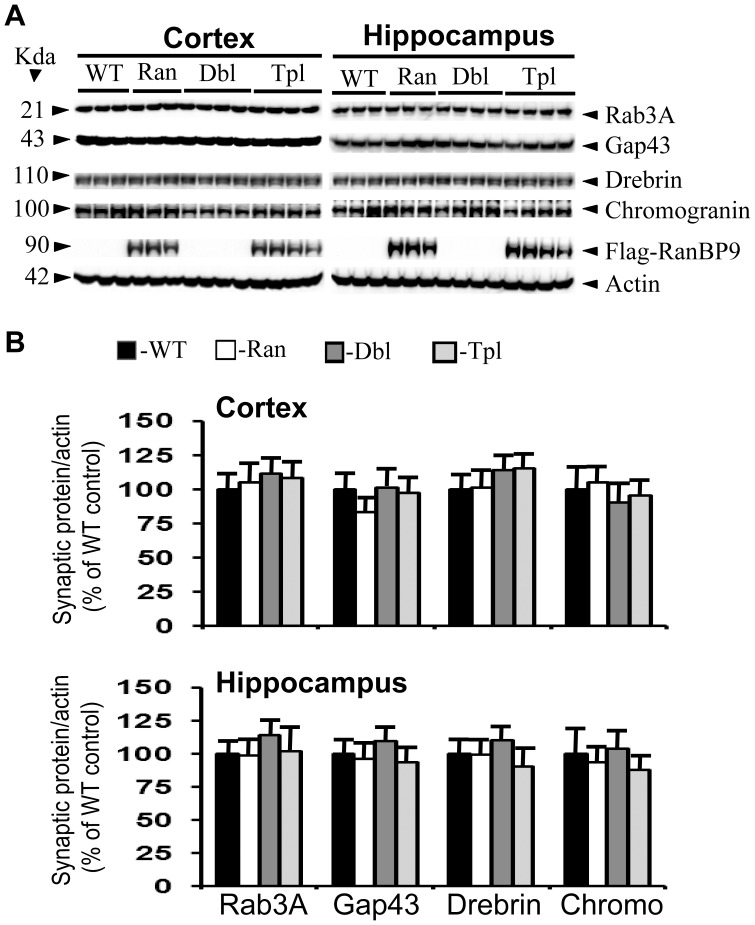
RanBP9 overexpression does not alter synaptic protein levels in the cortex and hippocampus at 4- months of age. **A:** Shows an immunoblotting analysis of rab3A, gap43, drebrin, chromogranin and the house keeping gene actin in brain samples from cortex and hippocampus. Brain homogenates from RanBP9 transgenic (Ran), APΔE9 double transgenic (Dbl), APΔE9/RanBP9 triple transgenic (Tpl) and age-matched wild-type (WT) control mice at 4-months of age were subjected to SDS-PAGE electrophoresis and probed with their respective antibodies. Flag specific monoclonal antibody detected flag-tagged exogenous RanBP9 in the RanBP9 single transgenic and APΔE9/RanBP99 triple transgenic mice only. Actin was used as loading control. The numbers on the left side indicate the molecular weights of each protein. **B:** Image J quantitation and normalization to actin levels showed no changes in the levels of any of the synaptic proteins at 4 months. The data are mean±SEM, n = 6 for WT and RanBP9 single transgenic, and n = 8 for APΔE9 and APΔE9/RanBP9 genotypes.

Although loss of synapses and synaptic proteins is well established in AD brains as well as mouse models of AD, the earliest ages when such anomaly begins to appear and also whether markers of AD progression have any influence on such a loss is unknown. Therefore we quantified the levels of two presynaptic and two post synaptic proteins in the APΔE9 double transgenic mice, and the APΔE9/RanBP9 triple transgenic mice. The generation and characterization of RanBP9 single transgenic and APΔE9/RanBP9 triple transgenic mice from our laboratory have been described [24], [28]. In order to restrict the expression of exogenous flag-tagged RanBP9 in the neurons only, we used thy-1 promoter. As there is selective loss of synapses in the cortical and hippocampal brain regions in both AD and in the mouse models of AD, we quantified the synaptic proteins in both the cortex and the hippocampus. The mean densitometric values of rab3A and gap43 were unaltered in both the cortex and the hippocampus of RanBP9 single transgenic, APΔE9 double transgenic and APΔE9/RanBP9 triple transgenic mice at 3 months (Fig. 1A & B) and 4-months (Fig. 2A & B) of age compared to age-matched wild-type (WT) controls. All protein levels were expressed with relative expression levels of actin which was used as loading control (panel 6 in all figures). Flag antibody detected the expression of exogenous flag-tagged RanBP9 only in the RanBP9 single transgenic and APΔE9/RanBP9 triple transgenic mice (panel 5 in all figures).

**Figure 3:**
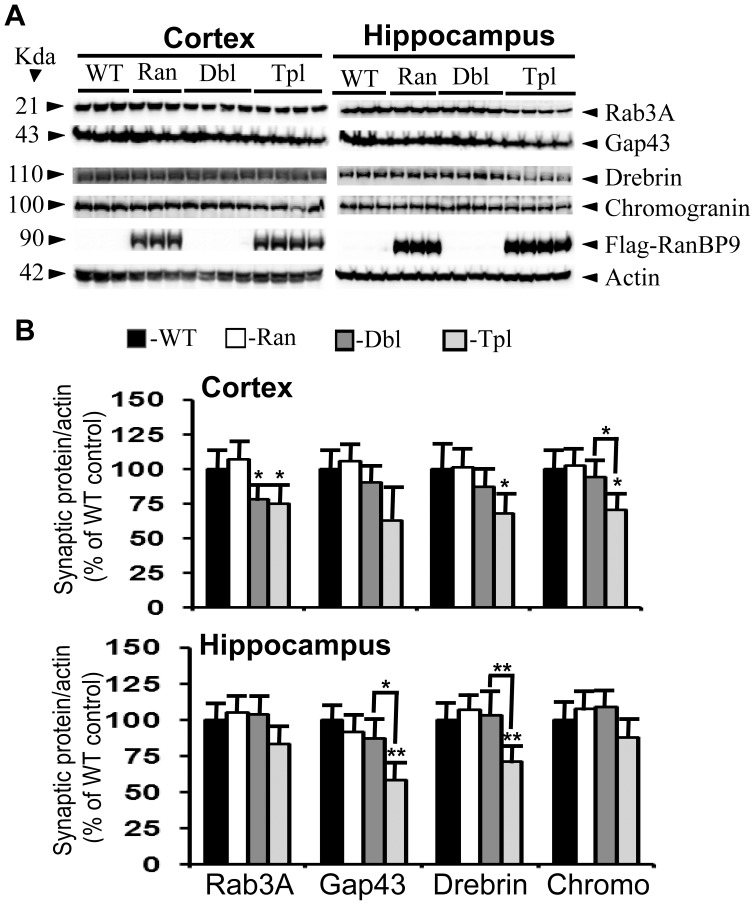
RanBP9 overexpression exacerbates reduction of synaptic protein levels at 5-months of age in the cortex and the hippocampus of APΔE9 mice. **A:** Brain homogenates were processed and synaptic proteins, flag-tagged RanBP9 and actin were detected as in legend to figure 1. **B:** ImageJ quantitation and normalization to actin levels revealed significant differences. Rab3A levels were reduced in the cortex by 22% and 25% respectively in the APΔE9 and APΔE9/RanBP9 mice compared to WT controls, but no changes were observed in the hippocampus. Gap43 levels were reduced by 44% only in the hippocampus of triple transgenic mice. Drebrin levels were reduced by 33% and 29% respectively in the cortex and the hippocampus. only in the APΔE9/RanBP9 mice compared to WT, but no change in APΔE9 mice versus WT or RanBP9 mice. Chromogranin levels were reduced only in the cortex by 30% in the triple transgenic mice. ANOVA followed by post-hoc Tukey’s test revealed significant differences. *, p<0.05, **, p<0.01 in APΔE9/RanBP9 or APΔE9 mice compared to WT mice. The data are mean±SEM, n = 6 for WT and RanBP9 mice, and n = 8 for APΔE9 and APΔE9/RanBP9 genotypes.

**Figure 4:**
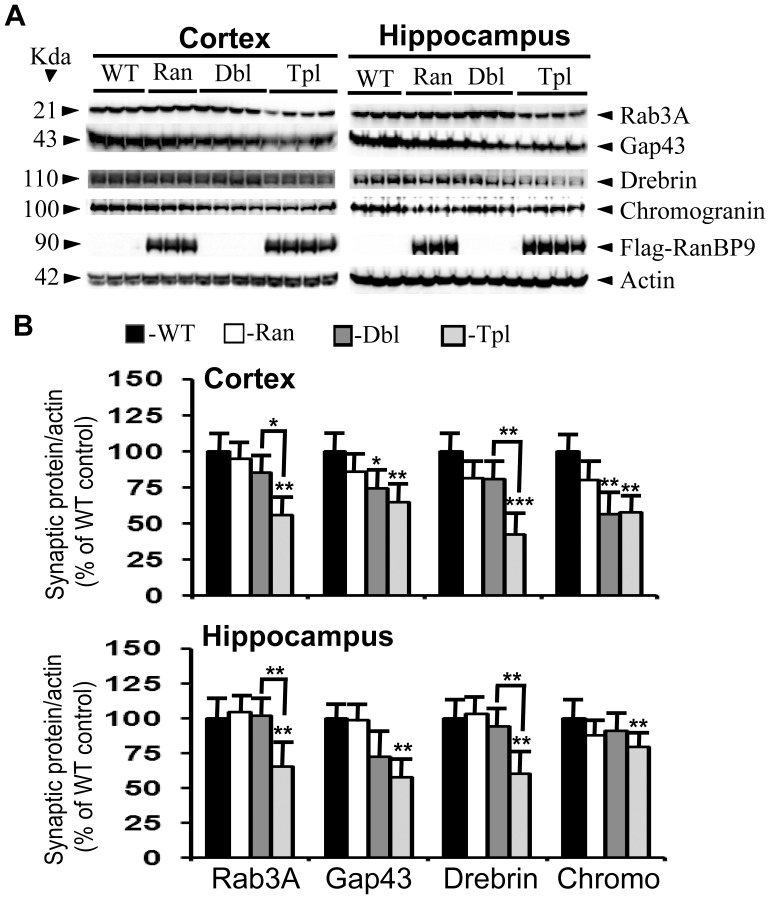
RanBP9 overexpression exacerbates reduction of synaptic protein levels at 6-months of age in the cortex and the hippocampus of APΔE9 mice. **A:** Brain homogenates were processed and synaptic proteins, flag-tagged RanBP9 and actin were detected as in legend to figure 1. **B:** ImageJ quantitation and normalization to actin levels revealed significant differences. Rab3A levels were further reduced by 30% and 36% in the cortex and hippocampus respectively in the triple transgenic mice compared to double transgenic mice. Similarly, drebrin levels were further reduced by 38% and 33% in the cortex and the hippocampus in the triple transgenic mice compared to double transgenic mice. Gap43 levels were reduced in the cortex by 36% in the triple transgenic mice and by 26% in the double transgenic mice compared to WT controls. In the hippocampus gap43 levels were reduced only in the triple transgenic mice by 43%. In the cortex, chromogranin levels were reduced by 44% in the double and by 44% in the triple transgenic mice, whereas in the hippocampus a 21% reduction was observed only in the triple transgenic mice. ANOVA followed by post-hoc Tukey’s test revealed significant differences. *, p<0.05, **, p<0.01, ***, p<0.001 in APΔE9/RanBP9 or APΔE9 mice compared to WT mice as indicated in the figure. The data are mean±SEM, n = 6 for WT and RanBP9 mice, and n = 8 for APΔE9 and APΔE9/RanBP9 genotypes.

At 5-months of age, rab3A levels were decreased in the APΔE9 (22%, p<0.05) and APΔE9/RanBP9 (25%, p<0.05) mice compared to WT controls only in the cortex but not hippocampus (Fig. 3A, panel 1 & 3B). Gap43 levels, on the other hand, decreased in the hippocampus but not the cortex in only APΔE9/RanBP9 mice (44%, p<0.01). Compared between APΔE9 and APΔE9/RanBP9 mice, there was 31% (p<0.05) further decrease in the levels of gap43 in the hippocampus due to RanBP9 overexpression (Fig. 3A panel 2& 3B,). By 6-months of age, the decreases in the levels of both rab3A and gap43 were more robust in the cortex as well as hippocampus. For rab3A, the decrease was 45% (p<0.01) and 35% (p<0.01) in the cortex and hippocampus respectively in the APΔE9/RanBP9 triple transgenic mice compared to WT controls, with no significant alterations in the APΔE9 mice (Fig. 4A, panel 1 and 4B). Of note, RanBP9 overexpression in the triple transgenic mice further decreased the levels of rab3A by 30% (p<0.05) and 36% (p<0.01) in the cortex and hippocampus respectively compared to APΔE9 mice (Fig. 4A, panel 1 and 4B). This suggests that RanBP9 overexpression exacerbates loss of rab3A in both the cortex and the hippocampus. Gap43 levels at 6-months of age were also significantly decreased by 36% (p<0.01) in the cortex and by 43% (p<0.01) in the hippocampus of APΔE9/RanBP9 triple transgenic mice compared to WT controls (Fig. 4A, panel 2 and 4B), with no significant alterations in the APΔE9 double transgenic mice. Thus, although there is evidence of dynamic changes in the levels of presynaptic proteins starting from 5- and 6-months of ages, the results suggests a consistently decreasing trend for both the proteins.

### RanBP9 Accelerates Loss of Postsynaptic Proteins, Drebrin and Chromogranin in the Cortex and the Hippocampus

Having confirmed significant loss of presynaptic proteins, we were interested to study the effect of RanBP9 overexpression in the APΔE9 mice on postsynaptic proteins as well since both pre and postsynaptic terminals are affected in AD. Thus, to ensure overall changes in the whole synaptic structure we examined two postsynaptic proteins, drebrin and chromogranin. Similar to changes in the levels of presynaptic proteins, the levels of drebrin (Fig. 1A, panel 3 and 1B) and chromogranin (Fig. 1A, panel 4 and 2B) were not significantly altered at 3-months of age in either the RanBP9 single transgenic, APΔE9 double transgenic or APΔE9/RanBP9 triple transgenic mice compared to age-matched WT controls in both the hippocampus and the cortex. At 4 months of age also, both drebrin (Fig. 2A, panel 3 and 1B) and chromogranin (Fig. 2A, panel 4 and 2B) were unaffected in any of the genotypes studied in both the cortex and the hippocampus. These results are consistent with those of presynaptic proteins and suggest that even under the condition of overexpression of RanBP9, a known molecular marker of AD progression, it is insufficient to alter any of the synaptic proteins.

However, by 5-months of age significant changes were noted in the levels of both drebrin and chromogranin. Drebrin levels were significantly reduced in the cortex by 33% (p<0.05) in the triple transgenic mice compared to WT controls (Fig. 3A, panel 3 and 3B). Neither the RanBP9 single transgenic nor APΔE9 double transgenic mice showed any alterations in the levels of drebrin in the cortex. In the hippocampus, the reduction was 29% (p<0.01) in the triple transgenic mice relative to WT controls. When the hippocampal drebrin levels were compared between APΔE9 and APΔE9/RanBP9 mice, the reduction was 32% (p<0.01) in the APΔE9/RanBP9 mice implying that RanBP9 overexpression worsens loss of drebrin protein (Fig. 3A, panel 3 and 3B). RanBP9 overexpression similarly worsened the loss of gap43 protein in the hippocampus but not in the cortex. Chromogranin levels, on the other hand were not altered in the hippocampus but showed reduced levels only in the cortex. Normalized levels of chromogranin was reduced by 30% (p<0.05) only in the APΔE9/RanBP9 triple transgenic mice compared to WT controls (Fig. 3A, panel 4 and 3B). A significantly further reduction (24%, p<0.05) was also noted between APΔE9 and APΔE9/RanBP9 genotypes.

Consistent with reductions in the levels of presynaptic proteins, the levels of postsynaptic protein were also worst affected relatively at 6-months of age in both the cortex and the hippocampus. Thus, in the cortex APΔE9/RanBP9 triple transgenic mice but not RanBP9 or APΔE9 mice showed a statistically significant reduction by 58% (p<0.01) in the levels of drebrin when compared to WT controls (Fig. 4A, panel 3 and 3B). RanbP9 overexpression further exacerbated the reduction by 38% (p<0.01) in the triple transgenic mice when compared to APΔE9 double transgenic mice (Fig. 3A, panel 3 and 3B). In the hippocampus the loss of drebrin protein was 40% (p<0.01) in the APΔE9/RanBP9 mice with no change in the levels either in the APΔE9 or RanBP9 mice (Fig. 3A, panel 3 and 3B) compared to WT controls. RanBP9 exacerbation in the hippocampus was 33% (p<0.01). However, chromogranin levels were reduced in the cortex in both the APΔE9 (44%, p<0.01) and APΔE9/RanBP9 (43%, p<0.01) mice when compared to WT controls. In the hippocampus, chromogranin protein level was reduced (21%, p<0.01) only in the APΔE9/RanBP9 triple transgenic mice (Fig. 3A, panel 4 and 3B). Thus at 6-months of age, both pre and postsynaptic proteins were more severely affected than other ages. This suggests that there is progressive reduction in the levels of synaptic proteins with respect to their age when compared to age-matched WT controls.

### Immunohistochemical Evidence for Loss of Synaptic Proteins in the Cortex and Hippocampus of APΔE9/RanBP9 Mice

**Figure 5:**
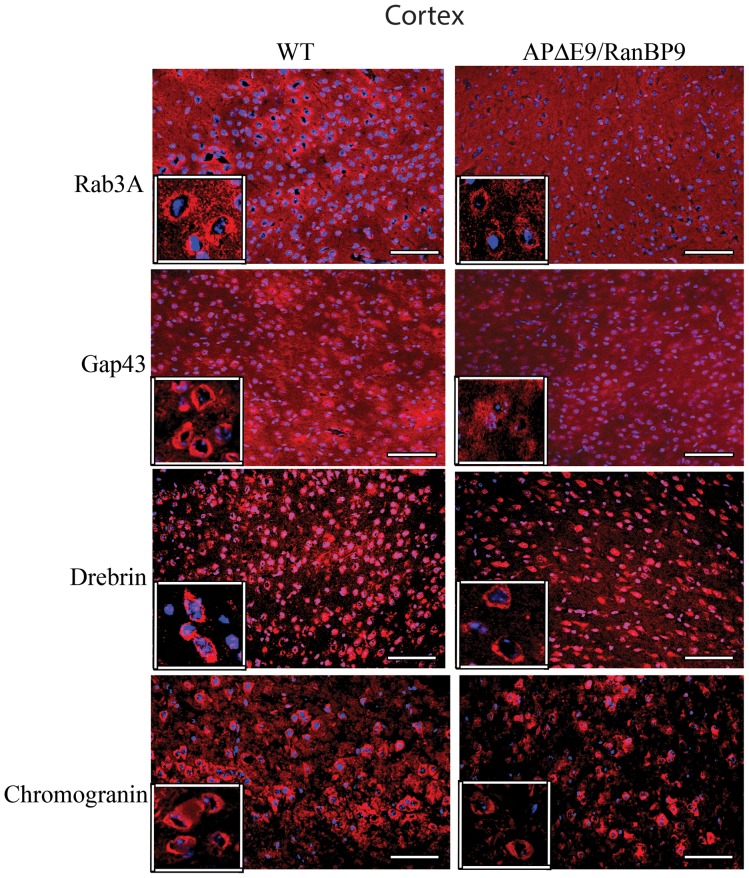
Immunohistochemical evidence for the reduced synaptic proteins at 6-months of age in the frontal cortex. Cortical brain sections from wild-type (WT) and APΔE9/RanBP9 triple transgenic mice were stained with antibodies against rab3a, gap43, drebrin and chromogranin. A qualitative difference is clearly seen with reduced immunoreactive puncta in the triple transgenic mice compared to WT brains for all the four synaptic proteins (red). The neuronal nuclei are stained blue. Scale bar: 100 µm.

**Figure 6:**
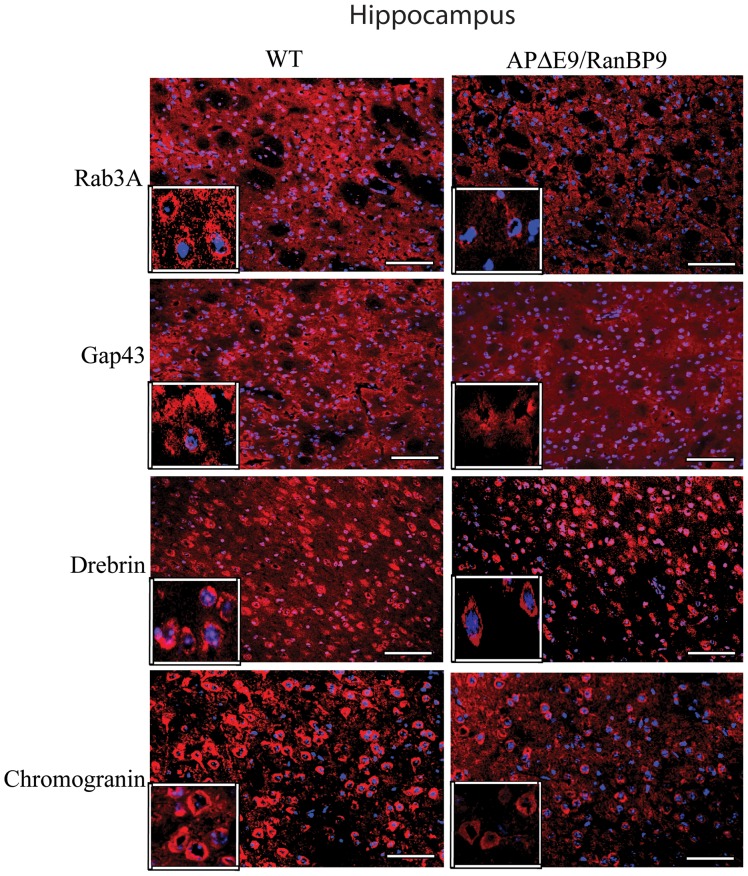
Immunohistochemical evidence for the reduced synaptic proteins at 6-months of age in the CA1 region of the hippocampus. Hippocampal brain sections from wild-type (WT) and APΔE9/RanBP9 triple transgenic mice were stained with antibodies against rab3a, gap43, drebrin and chromogranin. A qualitative difference is clearly seen with reduced immunoreactive puncta in the triple transgenic mice compared to WT brains for all the four synaptic proteins (red). The neuronal nuclei are stained blue. Scale bar: 100 µm.

To confirm loss of synaptic proteins by another method, we stained brain sections of WT and APΔE9/RanBP9 triple transgenic mice with specific antibodies against rab3a, gap43, drebrin and chromogranin. Because synaptic protein levels were most affected in the triple transgenic mice compared to WT at 6-months of age as revealed by immunoblots, we studied the staining pattern in these two genotypes only at 6-months of age. To assess the role of RanBP9 overexpression, we analyzed synaptic protein immunoreactivity in the CA1 region of the hippocampus and the frontal cortex. Although we did not quantify the fluorescence intensity, an apparent qualitative difference could be seen in the staining intensity in the triple transgenic mice versus WT controls in both the frontal cortex (Fig. 5) and the CA1 region of the hippocampus (Fig. 6) for all the four synaptic proteins studied. Thus immunohistochemical staining confirmed the immunoblot results and suggests that RanBP9 overexpression significantly reduces both the pre and postsynaptic proteins.

### RanBP9 Overexpression in APΔE9 Mice Significantly Reduces Levels of Aβ Oligomers

We previously showed that RanBP9 overexpression in APΔE9 mice reduced both the soluble Aβ monomers and amyloid plaques (24). To understand whether reduced synaptic proteins is due to RanBP9-induced alterations in the levels of Aβ oligomers, we quantified RIPA-soluble Aβ oligomers in the cortex of all four genotypes of mice using oligomer-specific antibody, A11. A11 has proven to recognize only amyloid oligomers of both mouse and human origin but not either amyloidogenic monomers or mature amyloid fibrils. At 3-months of age Aβ oligomers were almost undetectable in the WT and RanBP9 mice while in the APΔE9 and APΔE9/RanBP9 mice a faint band starts to appear suggesting that oligomers have not yet built up to any significant extent. At 4 and 5 months, a clear oligomer band is seen in both the APΔE9 and APΔE9/RanBP9 mice, but quantification revealed no significant differences among them.

**Figure 7:**
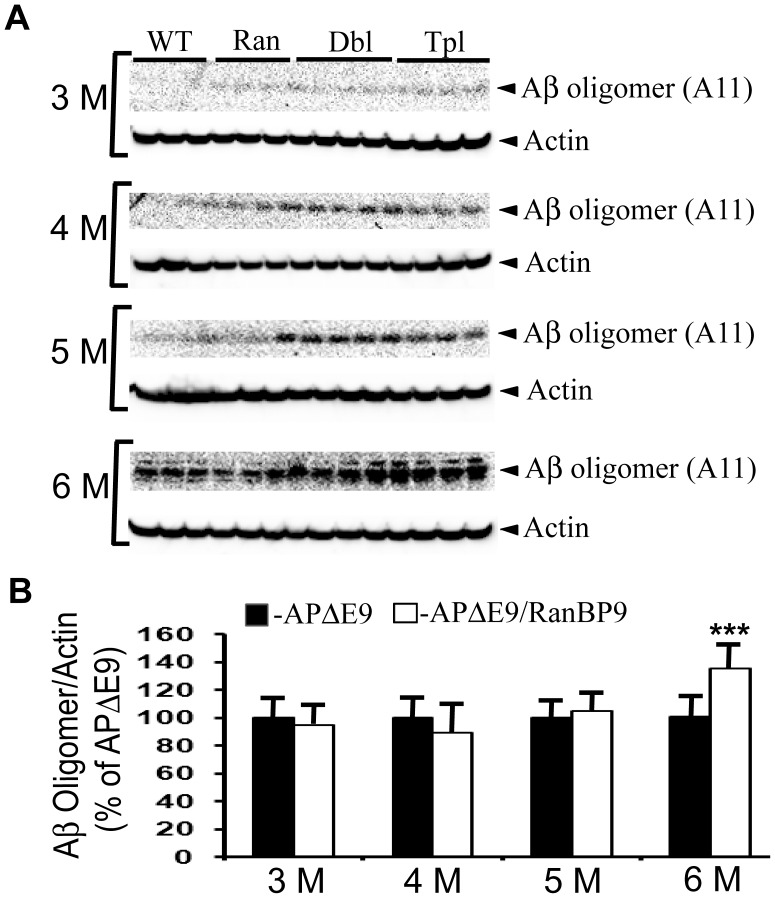
RanBP9 overexpression in APΔE9 mice increases Aβ oligomer levels at 6-months of age in the cortex. **A:** Proteins extracted from brains of wild-type (WT), RanBP9 single transgenic (Ran), APΔE9 double transgenic (Dbl) and APΔE9/RanBP9 triple transgenic mice (Tpl) of 3, 4, 5 and 6-months of age were homogenized in RIPA buffer and subjected to SDS electrophoresis and subsequently probed with anti-oligomer antibody, A11. **B:** For quantification of oligomers by image j, the data from WT and RanBP9 single transgenic mice were not considered since the levels were insignificant. Protein levels were normalized to actin and data expressed as percentage change. Student’s t-test analysis revealed significant increase in oligomer levels only at 6-months of age. Data are ±SEM, n = 3 for each of WT and Ran and 4 for each of APΔE9 and APΔE9/RanBP9 genotypes. ***, p<0.001 in the APΔE9/RanBP9 mice compared to APΔE9 mice.

At 6 months, oligomer levels were increased by 35% (p<0.001) in the APΔE9/RanBP9 mice compared to APΔE9 mice (Fig. 7A & B). Thus RanBP9 also increases levels of Aβ oligomers which may be responsible for the decreased levels of synaptic proteins.

## Discussion

In AD, the degeneration of synapses and neurons occurs in selective regions of the brain, including the frontal and parietal cortices [29], [30] and the hippocampus [31], which is the leading cause of cognitive impairment in AD. But the molecular mechanisms responsible for such a loss of synapses and more importantly precisely at what age such a loss of synapses occurs are not yet fully understood. Transgenic mouse models provide great opportunities to address such issues. We previously demonstrated that at 12-months of age, RanBP9 overexpression led to significant reductions in the levels of synaptophysin and PSD95 [23] and learning deficits [25]. In a more recent study [28], we confirmed significant reductions in the levels of spinophilin, a marker of dendritic spines in both the hippocampus and cortex at 12-months of age when RanBP9 was overexpressed in APΔE9 mice. The present study was primarily designed to identify the earliest possible age when loss of other synaptic proteins begins to appear in the APΔE9 mice under RanBP9 overexpression conditions.

The results consistently revealed that synaptic proteins are unaltered at 3- and 4-months of age in both the cortex and the hippocampus. Since we measured both the pre and postsynaptic proteins, the results imply that the synaptic structure as a whole is intact at these ages. At 5-months of age a trend towards significant reductions starts to appear. Interestingly, the reductions were not uniform in both the cortex and the hippocampus at 5-months of age. While rab3A and chromogranin were affected only in the cortex, gap43 was affected only in the hippocampus. Only drebrin protein levels were reduced in both the brains regions studied. Given the functions of synaptic proteins in neurotransmitter vesicle trafficking, docking and fusion to the synaptic membrane, it is not surprising that loss of synaptic proteins in AD is correlated with clinical symptoms [32] and that such a loss of synaptic proteins is brain region-specific [33]. It is conceivable that the reductions in the levels of synaptic proteins simply reflect the loss of synapses and that AD pathology may not be acting directly on these proteins. While such region specific loss of proteins is known for a long time in AD, the exact reason for such a differential vulnerability is not known. Regional differences in the neuronal activity, energy consumption and expression of specific molecules might play crucial roles in the regional vulnerability. Synaptic proteins play critical role in synaptic plasticity, which is thought to underlie learning and memory. The differential reductions in the levels of synaptic proteins at 5 months of age probably represent highly dynamic and transition state of the changes at this particular age because even by 6-months, the reductions were uniform for all the synaptic proteins in both the cortex and the hippocampus. In contrast to presynaptic markers, the postsynaptic structures have been less studied in both AD brains and the mouse models. As a major F-actin binding protein which is abundantly found in the dendritic spines, inclusion of drebrin in the present study is of major significance. Also, the present results suggest that RanBP9 affects both the pre and postsynaptic components of the synaptic structure as early as 6-months of age.

APΔE9 mice display about 50% reduction in the levels of synaptophysin as estimated by unbiased stereology in the hippocampus of 7 months old mice [34]. In the present study we found 43% reductions in the levels of only chromogranin in the cortex of APΔE9 mice at 6-months of age. Significantly, the expression of RanBP9 was sufficient to induce significant reductions of all four synaptic proteins in both the cortex and the hippocampus. Using another line of double transgenic mice expressing both APP and PS1 mutants similar to APΔE9 mice, Rutten et al., [35] also showed about 33% reduction in synaptophysin levels in the hippocampus. However no data is available for other synaptic markers in this model to our knowledge. Therefore direct comparisons can’t be made. Nevertheless, our carefully done study has revealed for the first time that both the pre and postsynaptic proteins can be significantly reduced by overexpressing RanBP9. Because RanBP9 levels are increased in AD brains [24], it is possible that RanBP9 is responsible for the reduced synapses and synaptic proteins in AD. Although at present we have no clue on how RanBP9 levels are increased in AD brains, in future studies we will investigate the contribution of miRNAs and epigenetic factors which are known to regulate many key genes. Thus RanBP9 accelerates the synaptic protein deficits to earlier ages with more robust reductions with respect to increasing age. Since RanBP9 reduces both pre and postsynaptic proteins, it also indicates that RanBP9 induces gross changes in the synaptic structure.

The present results are consistent with many documented properties of RanBP9. RanBP9 not only increases Aβ and amyloid plaques [22], [23], but also induces significant reductions in the levels of sAPPα [22]. Defective APP processing resulting in increased Aβ generation and most importantly decreased sAPPα levels is suggested to play crucial role in reduced synapses. A number of studies have shown that sAPPα exhibits neurotropic properties [36], [37]. A more recent study clearly demonstrated that application of sAPPα but not sAPPβ in the conditioned medium significantly restored loss of spines and dendritic branches in neurons cultured from APP−/− mice [38], suggesting that reduced sAPPα levels seen in AD patients might actually be responsible for the loss of synapses. Thus RanBP9-induced reduction in sAPPα levels or increased Aβ oligomer levels observed in the present study is likely at least partly responsible for the presently observed loss of synaptic proteins. Such loss of synaptic proteins at 5- and 6-months of age is responsible for the recently observed learning and memory deficits at the same ages in the APΔE9/RanBP9 triple transgenic mice [39]. Moreover, RanBP9 overexpression significantly increased amyloid plaque burden in the APΔE9 mice starting as early as 5-months of age but not at 3- or 4-months of age [39]. Thus there is excellent correlation between the loss of synaptic proteins learning and memory deficits and increased APP metabolism at the same ages. We also recently demonstrated that RanBP9 retards the anterograde transport of mitochondria, consequently leading to reduced synaptic mitochondrial activity [40]. Treatment of primary cortical neurons derived from RanBP9 transgenic mice with Trolox, which inhibits generation of reactive oxygen species (ROS) such as superoxide restored the anterograde transport of mitochondria toward synapses and the corresponding synaptic activity. In fact, direct measurement of mitochondrial activity in the synaptosomes derived from RanBP9 transgenic mice showed significant reductions [40]. When RanBP9 was overexpressed in the APΔE9 mice, it further exacerbated the deficits in mitochondrial activity, both under basal conditions and under ATP- and KCl-stimulated conditions [28]. Since synaptic terminals have high energy demands, reduced mitochondrial activity can be expected to compromise the viability and survival of synapses. As a result gradual loss of synapses might account for age-dependent loss of synaptic proteins observed in the present study.

The other indirect supporting evidence for RanBP9 to play crucial role at synapses comes from its subcellular localization and protein interactions. In primary neuronal cultures, RanBP9 is present throughout the neuron, especially in the entire network of neurites [23]. In the adult brain also, in addiction to soma, the dendritic processes also show the localization of significant amounts of RanBP9 [23] suggesting that RanBP9 has an important function at the synapses. Additionally, RanBP9 has been shown to interact with L1 receptor [41], integrin LFA-1 receptor [42] and Rho-GTPases [43], all of which are known to play crucial roles in the synaptic plasticity. Given the interaction of RanBP9 with many important proteins it is not surprising that RanBP9 reduces the synaptic proteins. But the most important observation in the present study is that RanBP9 accelerates the loss of synaptic proteins to earlier ages as early as 5-months of age when no abnormalities were found in the APΔE9 mice. Therefore future therapeutic interventions based on RanBP9 and RanBP9 signal transduction pathways may be an excellent approach to bring effective disease modifying therapy for AD.
