# Correction: RanBP9 Overexpression Accelerates Loss of Pre and Postsynaptic Proteins in the APΔE9 Transgenic Mouse Brain

**DOI:** 10.1371/journal.pone.0215713

**Published:** 2019-04-17

**Authors:** Hongjie Wang, Ruizhi Wang, Shaohua Xu, Madepalli K. Lakshmana

After publication of this article [1], concerns were raised about Figs 1–6.

- Fig 1A: The cortex Drebrin panel and the Hippocampus Drebrin panel appear to be identical.
- Fig 2A: The cortex Drebrin panel and the Hippocampus Drebrin panel appear to be identical.
- The Flag-RanBP9 Cortex panel in Figure 3A appears to be identical to the Flag-RanBP Hippocampus panel in Fig 4A.
- The Flag-RanBP9 Hippocampus panel in Figure 3A appears to be identical to the Flag-RanBP Cortex panel in Fig 4A.
- The Chromogenin Cortex panel in Fig 4A appears to be identical to Chromogenin Hippocampus panel in Fig 4A.
- Figs 5 and 6: Two pairs of ‘inset’ images appear to be identical to each other:
- Fig 5, Cortex Rab3A protein in transgenic mouse and Fig 6, Hippocampus, Rab3A protein in WT mouse
- Fig 5, Cortex, Gap43 protein in WT mouse and Fig 6, Hippocampus, Chromogranin protein in WT mouse
- The inset image in the Fig 5 APdeltaE9/RanBP9 GAP43 Cortex panel does not appear to correlate with the larger image

The authors acknowledge that they mistakenly re-used and mis-labeled some images during figure preparation that resulted in similarities between individual panels. Although the flag-RanBP9 blots in the Figure 3A cortex and Fig 4A hippocampus appear to be similar, the authors confirm that the blots represent independent experiments. The authors have provided replacement images for the following panels:

- Fig 1A Drebrin Hippocampus panel
- Fig 2A Drebrin Hippocampus panel
- Fig 4A Cortex flag-RanBP9 panel
- Insets in Fig 5 for Rab3A-APdE9/RanBP9, Gap43-WT, and insets in Fig 6 for Rab3A-WT, Chromogranin-WT

**Fig 1:**
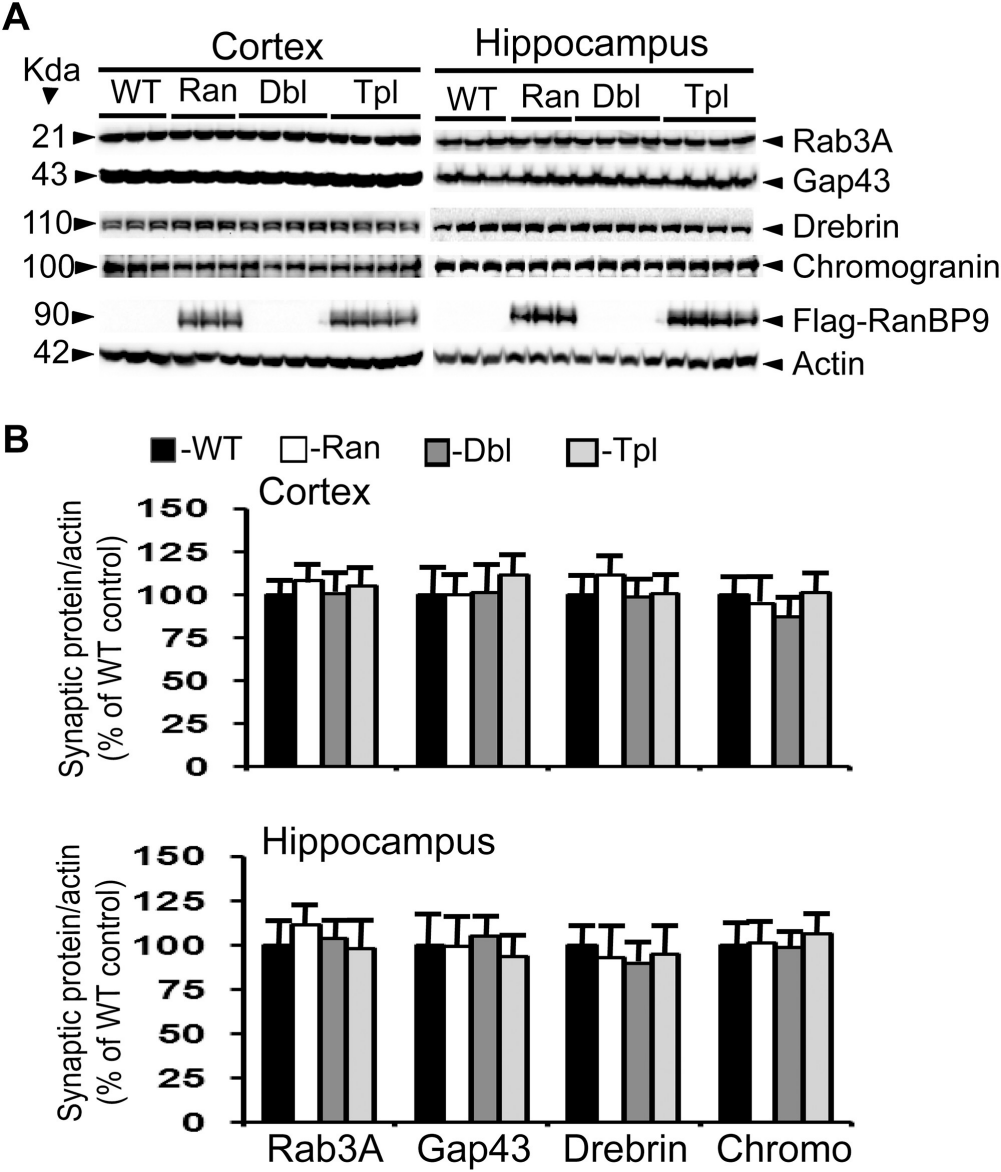
RanBP9 overexpression does not alter synaptic protein levels in the cortex and hippocampus at 3- months of age. A: Shows an immunoblotting analysis of rab3A, gap43, drebrin, chromogranin and the house keeping gene actin in brain samples from cortex and hippocampus. Brain homogenates from RanBP9 transgenic (Ran), APΔE9 double transgenic (Dbl), APΔE9/RanBP9 triple transgenic (Tpl) and age-matched wild-type (WT) control mice at 3-months of age were subjected to SDS-PAGE electrophoresis and probed with their respective antibodies. Flag specific monoclonal antibody detected flag-tagged exogenous RanBP9 in the RanBP9 single transgenic and APΔE9/RanBP99 triple transgenic mice only. Actin was used as loading control. The numbers on the left side indicate the molecular weights of each protein. B: Image J quantitation and normalization to actin levels showed no changes in the levels of any of the synaptic proteins at 3 months. The data are mean±SEM, n = 3 for WT and RanBP9 single transgenic, and n = 4 for APΔE9 and APΔE9/RanBP9 genotypes.

**Fig 2:**
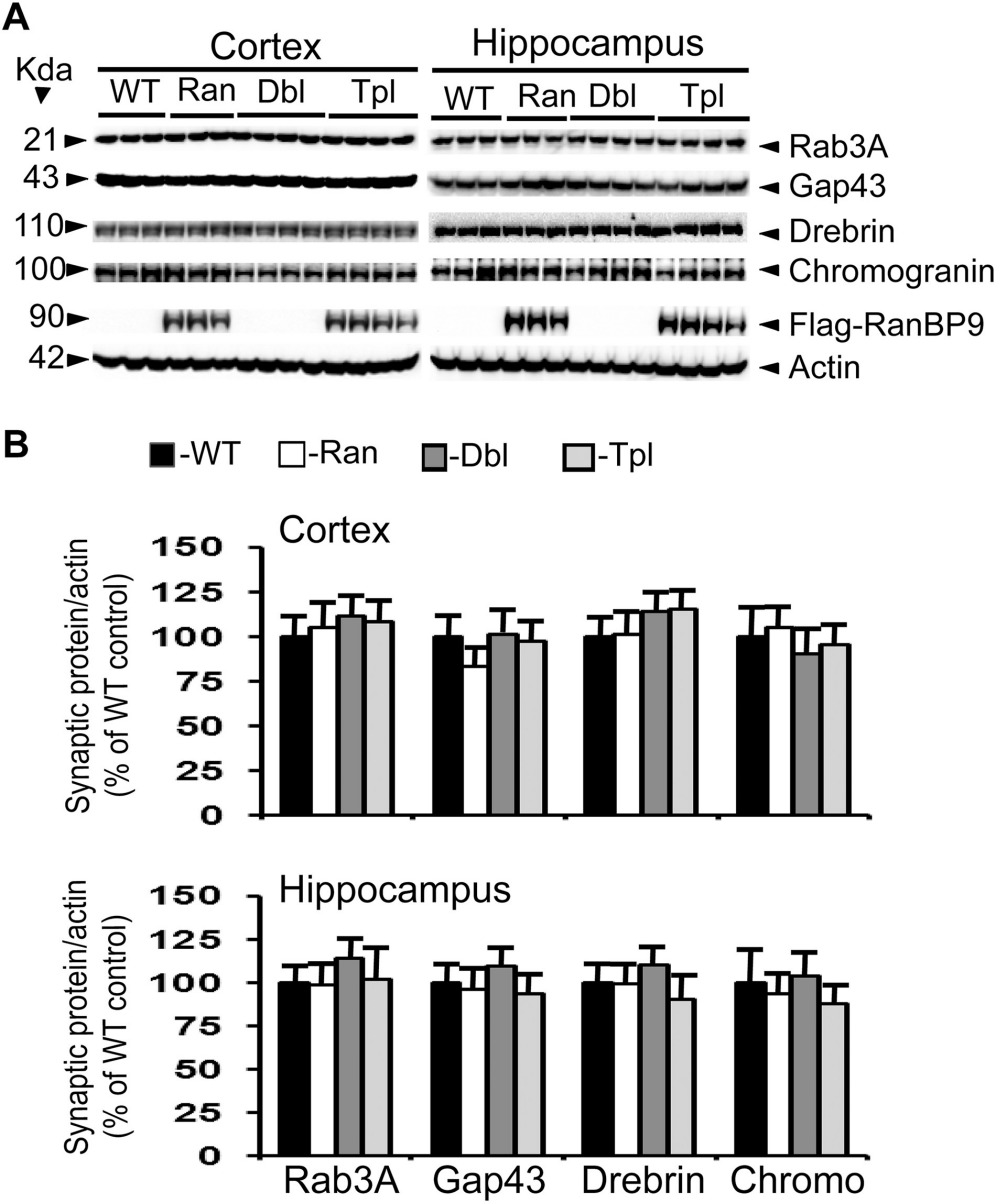
RanBP9 overexpression does not alter synaptic protein levels in the cortex and hippocampus at 4- months of age. A: Shows an immunoblotting analysis of rab3A, gap43, drebrin, chromogranin and the house keeping gene actin in brain samples from cortex and hippocampus. Brain homogenates from RanBP9 transgenic (Ran), APΔE9 double transgenic (Dbl), APΔE9/RanBP9 triple transgenic (Tpl) and age-matched wild-type (WT) control mice at 4-months of age were subjected to SDS-PAGE electrophoresis and probed with their respective antibodies. Flag specific monoclonal antibody detected flag-tagged exogenous RanBP9 in the RanBP9 single transgenic and APΔE9/RanBP99 triple transgenic mice only. Actin was used as loading control. The numbers on the left side indicate the molecular weights of each protein. B: Image J quantitation and normalization to actin levels showed no changes in the levels of any of the synaptic proteins at 4 months. The data are mean±SEM, n = 3 for WT and RanBP9 single transgenic, and n = 4 for APΔE9 and APΔE9/RanBP9 genotypes.

**Fig 4:**
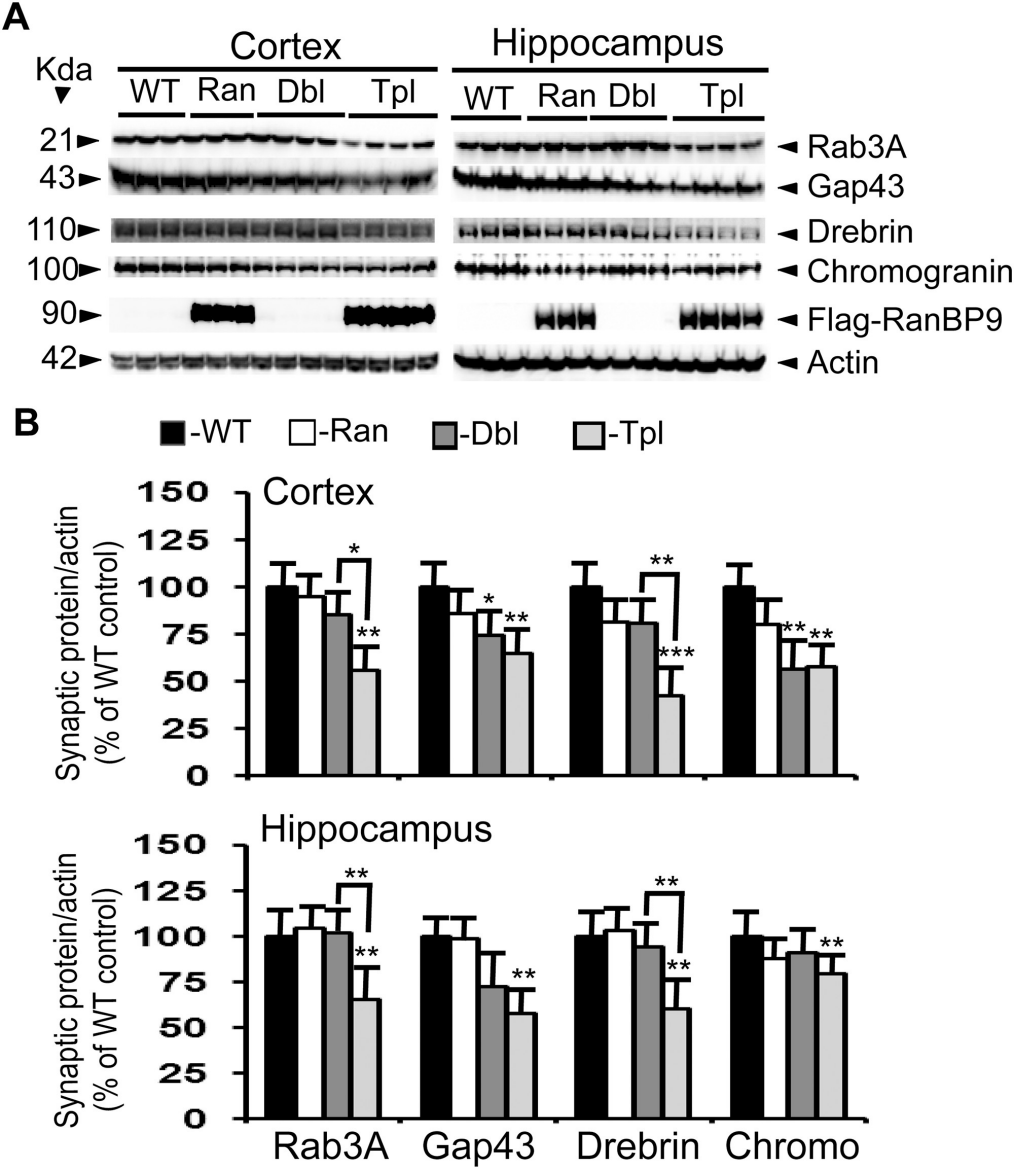
RanBP9 overexpression exacerbates reduction of synaptic protein levels at 6-months of age in the cortex and the hippocampus of APΔE9 mice. A: Brain homogenates were processed and synaptic proteins, flag-tagged RanBP9 and actin were detected as in legend to Fig 1. B: ImageJ quantitation and normalization to actin levels revealed significant differences. Rab3A levels were further reduced by 30% and 36% in the cortex and hippocampus respectively in the triple transgenic mice compared to double transgenic mice. Similarly, drebrin levels were further reduced by 38% and 33% in the cortex and the hippocampus in the triple transgenic mice compared to double transgenic mice. Gap43 levels were reduced in the cortex by 36% in the triple transgenic mice and by 26% in the double transgenic mice compared to WT controls. In the hippocampus gap43 levels were reduced only in the triple transgenic mice by 43%. In the cortex, chromogranin levels were reduced by 44% in the double and by 44% in the triple transgenic mice, whereas in the hippocampus a 21% reduction was observed only in the triple transgenic mice. ANOVA followed by post-hoc Tukey’s test revealed significant differences. *, p<0.05, **, p<0.01, ***, p<0.001 in APΔE9/RanBP9 or APΔE9 mice compared to WT mice as indicated in the figure. The data are mean±SEM, n = 3 for WT and RanBP9 mice, and n = 4 for APΔE9 and APΔE9/RanBP9 genotypes.

**Fig 5:**
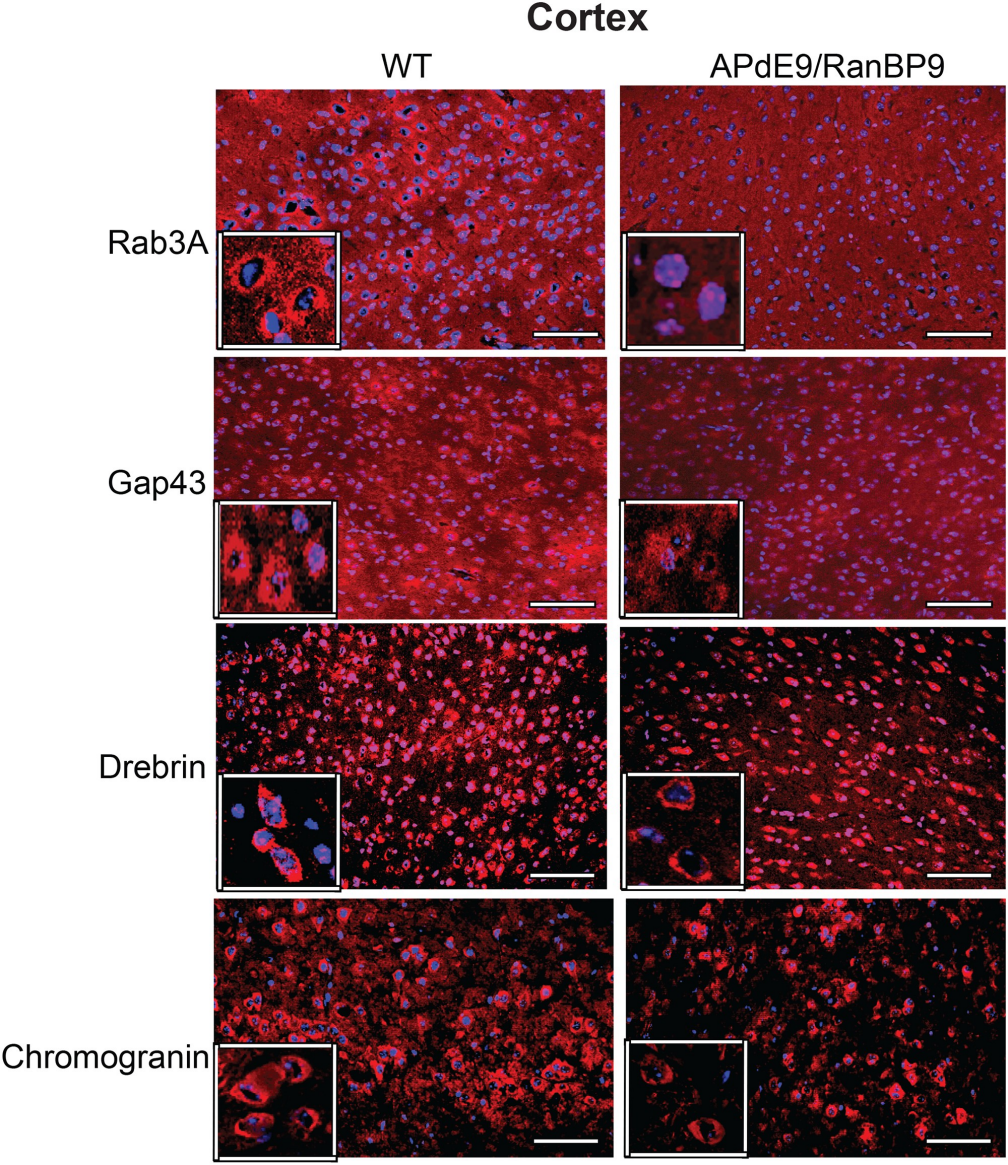
Immunohistochemical evidence for the reduced synaptic proteins at 6-months of age in the frontal cortex. Cortical brain sections from wild-type (WT) and APΔE9/RanBP9 triple transgenic mice were stained with antibodies against rab3a, gap43, drebrin and chromogranin. A qualitative difference is clearly seen with reduced immunoreactive puncta in the triple transgenic mice compared to WT brains for all the four synaptic proteins (red). The neuronal nuclei are stained blue. Scale bar: 100 μm.

**Fig 6:**
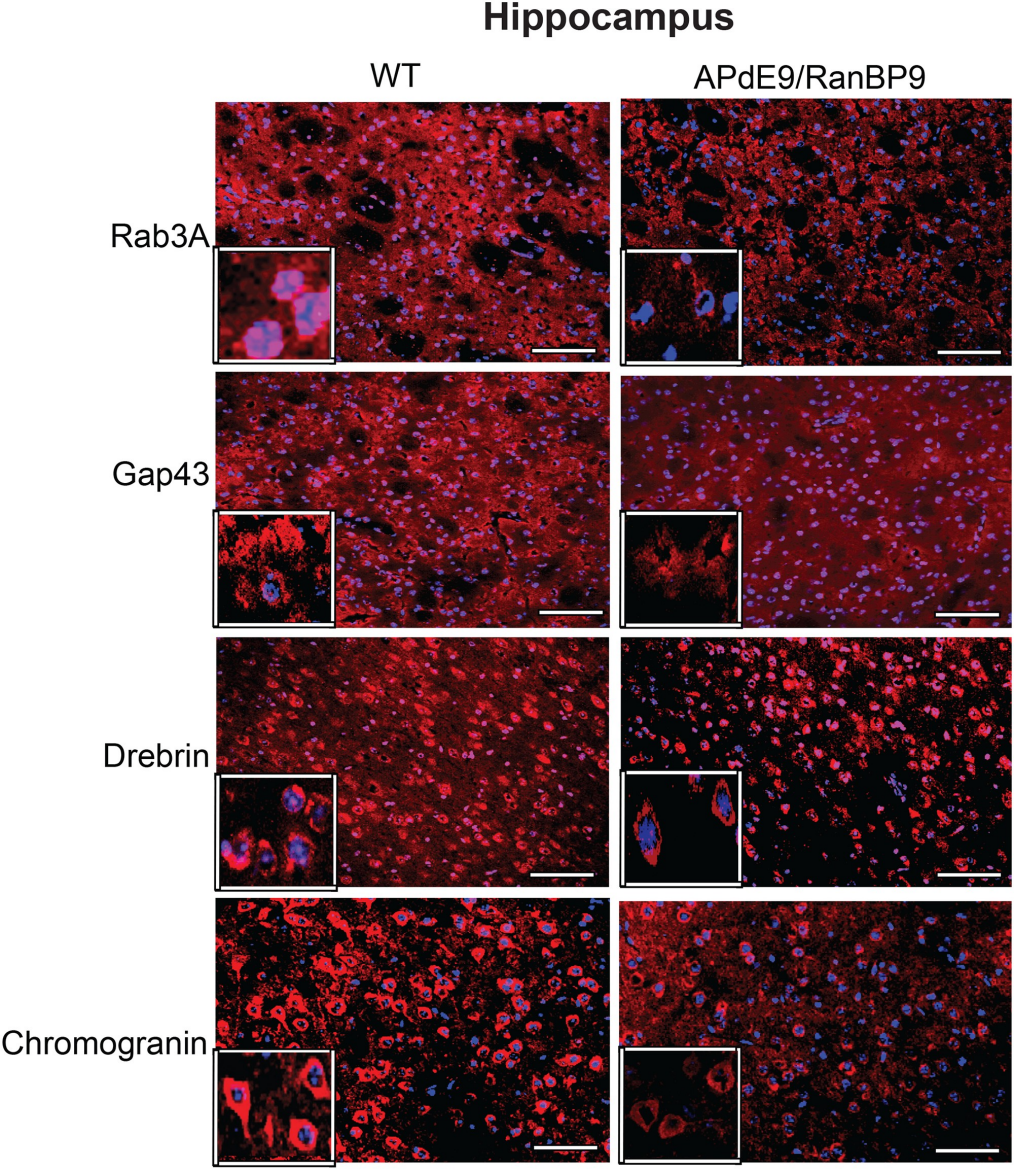
Immunohistochemical evidence for the reduced synaptic proteins at 6-months of age in the CA1 region of the hippocampus. Hippocampal brain sections from wild-type (WT) and APΔE9/RanBP9 triple transgenic mice were stained with antibodies against rab3a, gap43, drebrin and chromogranin. A qualitative difference is clearly seen with reduced immunoreactive puncta in the triple transgenic mice compared to WT brains for all the four synaptic proteins (red). The neuronal nuclei are stained blue. Scale bar: 100 μm.

An Academic Editor reviewed the updated figure(s) and underlying original data files and confirmed that they support the results and conclusions reported in the published article.

The authors apologize for the errors in the published article and confirm that all the primary data underlying other figures in the article are available in Figshare at the following link: https://figshare.com/s/7a05b8d2bebef5410bae. The authors were unable to locate the actin blot for hippocampus in Fig 1 because of accidental deletion of the original file.
